# Bilateral Femoral Insufficiency Fractures Likely Related to Long-Term Alendronate Therapy

**DOI:** 10.4061/2011/810697

**Published:** 2011-05-05

**Authors:** Ravindra Gudena, Jason Werle, Kelly Johnston

**Affiliations:** Department of Orthopedics, University of Calgary, 711B, 3607-49 ST NW, Calgary, Alberta, Canada T3A 2H3

## Abstract

Osteoporosis-related fractures are a major public health problem and one in two women and one in four men are affected with osteoporosis-related fractures. Alendronate (Fosamax) is one of the first bisphosphonates used to treat osteoporosis effectively. Recently, however, there is a concern regarding long bone insufficiency fractures related to long-term alendronate therapy. We report a case of bilateral femoral insufficiency fractures likely related to long-term alendronate therapy, the classic symptoms, signs, and treatment of these fractures.

## 1. Introduction

Osteoporosis-related fractures are a major public health problem mainly in postmenopausal women and the elderly. Osteoporosis affects an estimated of 75 million people in Europe, United States of America, and Japan [1]. There are several treatment options available including calcium, vitamin D, parathyroid hormone, strontium, and bisphosphonates. Since their introduction, bisphosphonates have been widely used in osteoporotic treatment. Alendronate (Fosamax, Merck & company, inc.) is one of the first bisphosphonates to be licensed and used. There are several studies showing the benefits of alendronate and other bisphosphonates. Recently, however, there is an emerging concern regarding long bone stress fractures secondary to long-term alendronate intake. We present a case of bilateral femoral shaft insufficiency fractures secondary to long-term alendronate therapy. 

## 2. Case Report

**Figure 1:**
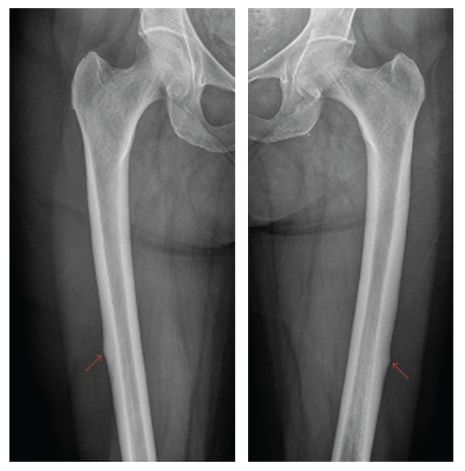
Radiograph of both femurs showing stress fracture and lateral cortical thickening.

**Figure 2:**
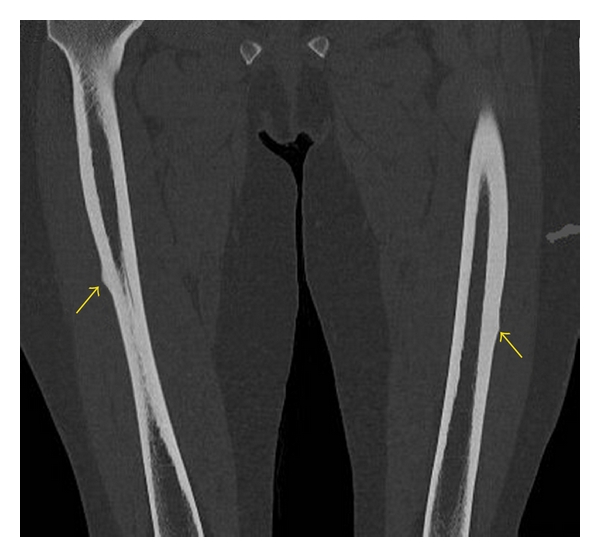
Computed tomography reveals bilateral femoral stress fractures.

**Figure 3:**
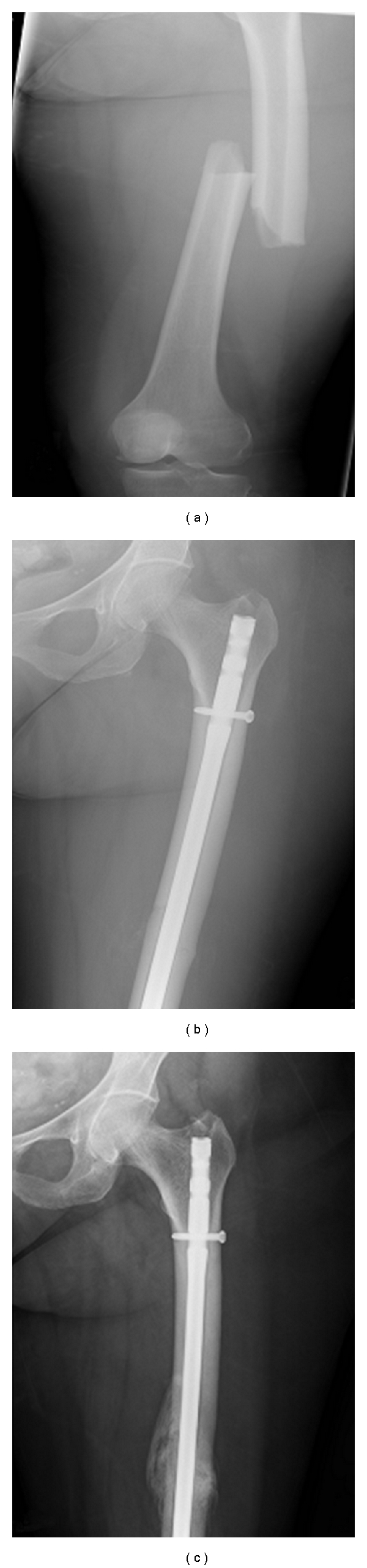
Fracture left femur at the level of stress fracture site, immediate postoperative, and three months postoperative radiographs.

A 74-year-old lady presented to orthopaedic clinic with a four-month history of bilateral thigh pain. She had no history of trauma. She had history of breast cancer treated with lumpectomy 10 years ago and has a history of well-controlled asthma, for which she did not require corticosteroid treatment. She had been receiving alendronate therapy for postmenopausal osteoporosis for 10 years. Radiographs revealed lateral cortical thickening of the mid-diaphysis of both femurs (Figure 1). The patient underwent a bone scan to rule out the possibility of metastases due to her previous history. The bone scan raised the possible diagnosis of a healing stress fracture. Computed tomography scan confirmed a stress fracture (Figure 2). She was given the advice of protective weight bearing and planned followup. Four days after consultation, she tripped and sustained a transverse fracture of the left femur and underwent uneventful femoral intramedullary nailing (Figures 3(a) and 3(b)). An endocrinology consultation was sought, and alendronate therapy was stopped. Bone mineral density scan was organized, and she was started on teriparatide treatment. Because there was no progressive right thigh pain, no further surgical management was offered beyond close followup. At four months postoperatively, the left femur fracture showed good radiological union (Figure 3(c)). There was still persistent pain in the right femur and a definite linear lucency in the lateral femoral cortex on plain radiographs. She underwent prophylactic intramedullary nailing of the right femur and had eventual complete relief of her symptoms.

## 3. Discussion

The lifelong risk of having a fracture related to osteoporosis is one in two for women and one in four for men. Prevention and treatment of osteoporosis is a major public health concern. Bisphosphonates have been widely used for treatment of osteoporosis. They are antiresorptive agents which act via inhibition of osteoclasts. As a consequence, bone formation coupled to resorption is also decreased resulting in an overall reduction of bone remodelling. Bisphosphonates also combine with calcium and become incorporated in the crystal structure of bone and retained within the skeleton for long periods of time. Therefore, they have the potential to exert efforts long after the treatment has been discontinued. Alendronate is the first bisphosphonate to be approved for the treatment and prevention of osteoporosis. Several previous studies have shown that alendronate reduces the risk of osteoporotic fractures and osseous resorption in high turnover bone disease. It has an excellent benefit to risk ratio for women with osteoporosis when used for 3-5 years. However, the benefit of continuing beyond five years is not established. 

In addition to other well-known side effects of bisphosphonates, there is a recent concern regarding the association of cortical insufficiency fractures and low-energy femoral fractures with long-term alendronate use. Several published case series and case reports suggest femoral shaft, and subtrochanteric femur fractures can be associated with long-term alendronate use [2–4]. Lenart et al. have shown a significant proportion of patients, in their study of low-energy sub-trochanteric and diaphyseal femur fractures, were on long-term bisphosphonates. Clinical and radiological features are almost unique in all of these reports [2]. The characteristic features include prodromal pain in the thigh for several months prior to the fracture (76% of the reported cases), bilateral fractures either sequential or simultaneous, and fractures with trivial trauma. The radiological features of these fractures are lateral cortical thickening, cortical spiking or beaking over the medial cortex, and transverse or short oblique fractures. 

Neviaser et al. reported in their study that 19 of the 25 patients on alendronate demonstrate a simple transverse fracture with a unicortical beak in an area of cortical hypertrophy. In contrast, this fracture pattern was seen in only one patient who was not being treated with alendronate, indicating that the fracture pattern is typical in alendronate users [3]. Our patient also presented with these classic symptoms and radiological signs. 

Femoral insufficiency fractures after prolonged alendronate therapy seldom heal spontaneously. Alendronate acts as a strong inhibitor of osteoclastic bone resorption which reduces the overall bone turnover. This has a negative effect on bone healing and is likely one of the reasons why many of the insufficiency fractures have delayed healing. Since alendronate inhibits osteoclastic remodeling, endochondral fracture repair is the preferred method of fracture healing. Intramedullary reconstruction with full-length nails accomplishes this goal and protects the entire femur. Locking plates preclude endochondral repair and are not recommended as the method of fixation [4]. Sayed-Noor and Sjödén reported a case series of delayed union even after internal fixation of a femoral insufficiency fracture in a patient who was on long-term alendronate [5]. Visekruna et al. reported no radiographic evidence of union out as far as 22 months postoperatively [6]. Capeci and Tejwani and Ha et al. reported union at an average of four and five months, respectively, after internal fixation [7, 8]. In our case, the left femur fracture was treated with a reamed intramedullary nail with union at four months. Because of the nonprogressive pain from the right femur stress fracture, close observation with protective weight-bearing was initially chosen but proved to be insufficient despite stopping alendronate. Ultimately, prophylactic intramedullary nailing was performed four months after with immediate symptom resolution. We support the view of Capeci and Tejwani and Ha et al. to treat even the incomplete insufficiency fractures with internal fixation. As well, we feel clinicians should not be lured into conservative management by the radiographic features of focal cortical hypertrophy. Dietary calcium and vitamin D status should be assessed, and adequate supplementation should be prescribed. Teriparatide therapy can improve or hasten healing of these fractures [6]. 

Although there is strong evidence supporting short-term alendronate therapy to reduce the risk of osteoporotic fractures, there is not enough evidence to suggest benefit in long-term use beyond five years. The continuation of alendronate therapy after five years should be tailored to individual patients after appropriate followup and the need for ongoing therapy reassessed annually [4]. Any patients with prodromal symptoms such as thigh pain should be investigated with routine bilateral radiographs of the femur looking for stress reaction, and if necessary, advanced imaging-like bone scan or magnetic resonance imaging. This is not the first case report on insufficiency diaphyseal fractures related to long-term alendronate therapy. However, this is a case report with characteristic features and bilateral involvement which highlights the importance of evaluating patients on long-term alendronate therapy and recommends early prophylactic internal fixation of these fractures.

## 4. Key Points

- Long-term alendronate therapy is likely associated with femoral insufficiency fractures.
- Patients on more than 5 years of alendronate therapy should be reevaluated annually regarding continuation of treatment and need close observation.
- Patients with thigh pain on alendronate therapy should be investigated for femoral stress fractures.
- Insufficiency fractures related to alendronate therapy seldom heal without internal fixation. Teriparatide can hasten healing of these fractures.

## 5. Conflict of Interests

The authors declare that they have no conflict of interests.
